# Rare gene variants in a patient with azathioprine-induced lethal myelosuppression

**DOI:** 10.1007/s00277-017-3112-9

**Published:** 2017-08-22

**Authors:** Xiaoxue Yang, Heng Xu, Jun Yang, Li Yang

**Affiliations:** 1 Department of Gastroenterology and Hepatology, West China Hospital, Sichuan University, Chengdu, Sichuan People’s Republic of China; 2 Department of Laboratory Medicine, Precision Medicine Center, State Key Laboratory of Biotherapy and Precision Medicine Key Laboratory of Sichuan Province, West China Hospital, Sichuan University and Collaborative Innovation Center, Chengdu, Sichuan China; 3 Department of Pharmaceutical Sciences, St. Jude Children’s Research Hospital, Memphis, TN USA

**Fig. 1:**
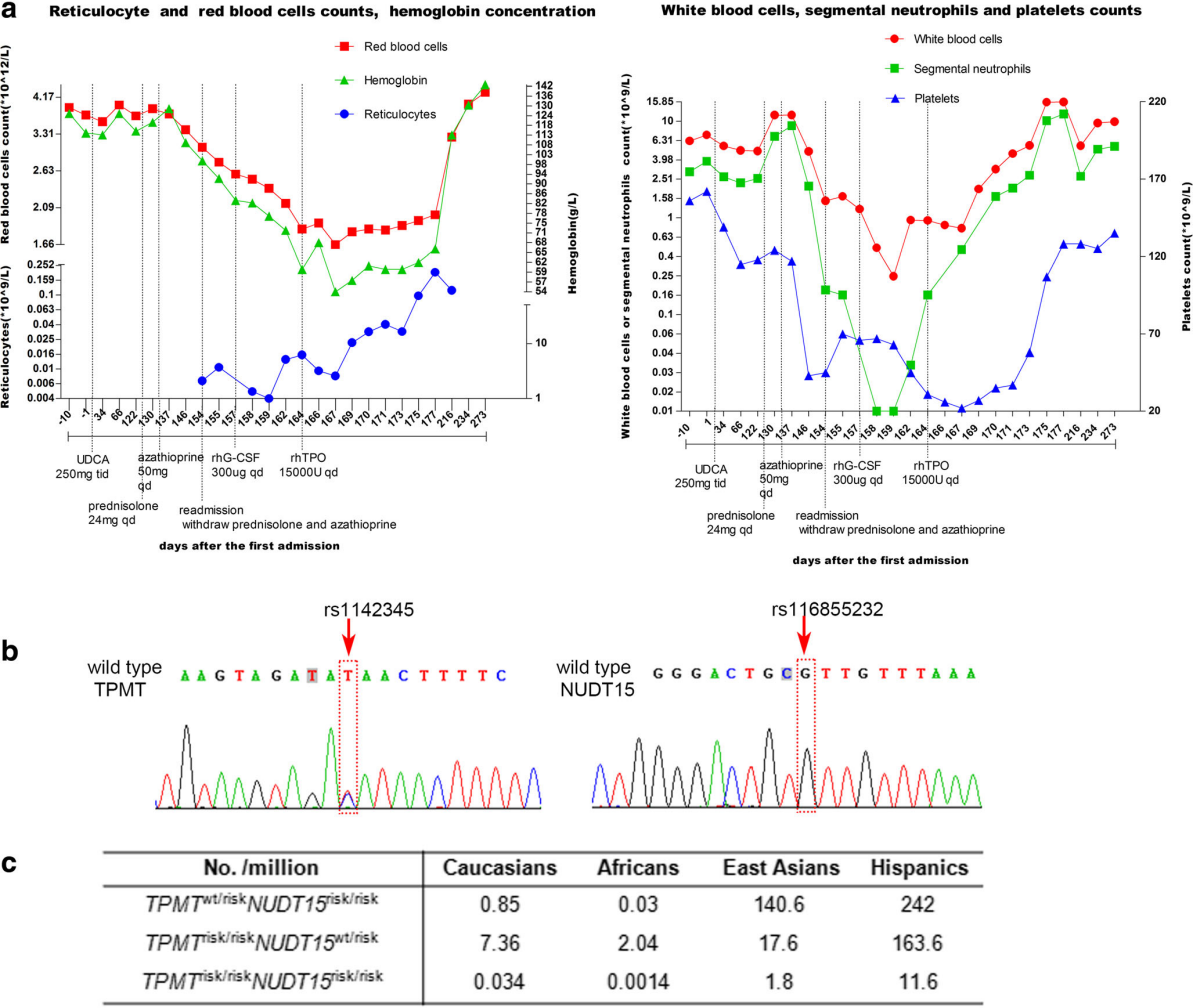
The changes of blood cells and gene testing of the patient. **a** The changes of white blood cells, neutrophilic granulocytes, red blood cells, reticulocytes, hemoglobin, and platelet in the clinical course. **b** The frequency of concurrence of rs1142345 and rs116855232 risk allele (at least homozygous for one SNP) in diverse ethnicities. **c** The *NUDT15* and *TPMT* gene variants of the patient. The GenBank accession number of the *TPMT* and *NUDT15* DNA are NG_012137.2 and NG_047021.1. Abbreviations: TPMT = thiopurine S-methyltransferase; NUDT15 = nudix hydrolase 15; UDCA = ursodeoxycholic acid; rhG-CSF = Human Granulocyte Colony Stimulating Factor; rhTPO = Recombinant human thrombopoietin

Dear Editor,

A 46-year-old Chinese male patient was diagnosed with autoimmune hepatitis—primary biliary cholangitis overlap syndrome at the first admission on Apr 22nd 2016. He was treated with ursodeoxycholic acid, followed by prednisolone and azathioprine (Fig. 1a). He was admitted again with fever and sore throat on Sept 15th, showing visible throat swelling. Notably, a sharp drop of blood cells was observed, reaching the diagnostic criteria of grade 4 myelosuppression (Fig. 1a), with 0.18 × 10^9^/L for neutrophilic granulocytes. Azathioprine and prednisolone were stopped immediately, while human granulocyte colony-stimulating factor and recombinant human thrombopoietin were prescribed to increase blood cells. However, the patient still suffered a life-threatening progressive decrease of blood cells. The lowest level of neutrophilic granulocytes, reticulocytes, and white blood cells reached 0.01 × 10^9^/L, 0.0040 × 10^12^/L, 0.25 × 10^9^/L, respectively, on Sept 23rd, while the hemoglobin, platelets, and red blood cells dropped to the lowest level at 54 g/L, 22*10^^9^/L, and 1.66*10^^12^/L, respectively, on Oct 1st (Fig. 1a). Moreover, diffuse rash and severe alopecia were observed. Serious infections (Pseudomonas aeruginosa infection) also occurred during hospitalization. The blood counts recovered far more slowly than expected and it returned to normal until 25 days (Oct 10th) after therapy (Fig. 1a). We attempted to genotype the SNPs of *NUDT15* (rs116855232) and *TPMT* (rs1142345) of this patient and found that this patient has *NUDT15* ^risk/risk^ *TPMT* ^wt /risk^ genotype (Fig. 1b).

The frequency of rs1142345 and rs116855232 exhibits largely ethnic differences. According to the large genetic variant screening in diverse ethnicities, the risk allele frequency of rs1142345 is high in Caucasians (4%), Hispanics (4.8%), and Africans (5.4%) but low in East Asians (1.3%), while that of rs116855232 is high in East Asians (10.4%) and Hispanics (7.1%) but rare in Caucasians (0.46%) and Africans (0.07%) (http://exac.broadinstitute.org/). Therefore, concurrence of rs1142345 and rs116855232 risk allele (at least homozygous for one SNP) is pretty rare (Fig. 1c), which have never been reported in thiopurine treatment before. Indeed, the frequency of the genotype we reported is ~ 140.6 per million in East Asians. The variant can induce activity deficiency of both TPMT and NUDT15 enzymes, thus reducing the degradation of 6-thioguanine nucleotides through different mechanisms [1]. Not surprisingly, the patient suffered severe life-threatening myelosuppression with very early occurrence and long duration after azathioprine treatment. Therefore, lower thiopurine dosage should be used in patients with such genotype than those with *NUDT15* ^risk/risk^ or *TPMT* ^risk /risk^ alone [2]. However, guidelines for autoimmune liver diseases have not referred to *NUDT15* genetic examinations before azathioprine treatment. Given that *NUDT15* and *TPMT* are commercially available to be checked, more clinicians should be aware that it is important to detect both *TPMT* and *NUDT15* SNPs before thiopurine treatment in autoimmune liver diseases, especially in East Asians and Hispanics. Additionally, other less common SNPs in these two genes should be also considered, such as rs147390019 with an allele frequency of 1.7% in Hispanics [3].
